# Author Correction: Dietary restriction reveals sex-specific expression of the mTOR pathway genes in Japanese quails

**DOI:** 10.1038/s41598-024-63532-8

**Published:** 2024-06-04

**Authors:** Gebrehaweria K. Reda, Sawadi F. Ndunguru, Brigitta Csernus, Renáta Knop, James K. Lugata, Csaba Szabó, Levente Czeglédi, Ádám Z. Lendvai

**Affiliations:** 1 Department of Animal Science, Faculty of Agricultural and Food Sciences and Environmental Management, Institute of Animal Science, Biotechnology and Nature Conservation, University of Debrecen, 4032 Debrecen, Hungary; 2 Doctoral School of Animal Science, University of Debrecen, 4032 Debrecen, Hungary; 3 Department of Evolutionary Zoology and Human Biology, University of Debrecen, 4032 Debrecen, Hungary; 4 Department of Animal Nutrition and Physiology, Faculty of Agriculture and Food Sciences and Environmental Management, University of Debrecen, 4032 Debrecen, Hungary

Correction to: *Scientific Reports* 10.1038/s41598-024-58487-9, published online 09 April 2024

The original version of this Article contained an error in the Data Availability section where,

“All data generated or analysed during this study are included in this published article [and its Supplementary Information files]. Upon acceptance of the manuscript, the datasets will be uploaded into a public repository.”

now reads:

“All data analysed during this study are included in this published article on its Supplementary Information files.”

The original Article has been corrected.

